# The Nation’s First Publicly Recognized Overdose Prevention Centers: Lessons Learned in New York City

**DOI:** 10.1007/s11524-023-00717-y

**Published:** 2023-04-04

**Authors:** Rebecca E. Giglio, Shivani Mantha, Alex Harocopos, Nilova Saha, Jacqueline Reilly, Chelsea Cipriano, Maura Kennelly, Lisa Landau, Michael McRae, Dave A. Chokshi

**Affiliations:** 1 New York City Department of Health and Mental Hygiene, Queens 42-09 28Th St, New York, NY 11101 USA; 2 New York Health Foundation, New York, NY USA; 3 New York University Grossman School of Medicine, New York, NY USA

**Keywords:** Opioid overdose, Substance use, Supervised consumption, Overdose prevention centers

## Abstract

In November of 2021, multiple factors converged to create a window of opportunity to open overdose prevention centers (OPCs) at two existing syringe service programs (SSPs) in New York City (NYC). Political will exists in NYC, particularly toward the end of the de Blasio administration’s term, and the NYC Health Department worked to garner additional support from local and state elected officials given the dire need to address the overdose crisis. This coincided with readiness on the part of one of the NYC SSP providers, OnPoint NYC, to open and operate OPC services. Legal risks were assessed by both the city and the provider. This case study outlines the sequence of events that resulted in NYC supporting OnPoint to open the first two publicly recognized OPCs in the nation, including lessons learned to inform other jurisdictions considering offering such services.

## Introduction

On November 30, 2021, OnPoint NYC opened the first publicly recognized OPC in the nation at two of NYC existing syringe service programs (SSP). Building on a range of harm reduction, healthcare, and social services offered by SSPs, OPCs offer safe, hygienic spaces in which people can use pre-obtained drugs under the supervision of staff trained to respond to an overdose. Operating in more than 10 countries, data have demonstrated that supervised consumption facilities reduce harm for people who use drugs (e.g., transmission rates of infectious diseases) and prevent fatal overdoses. No deaths have ever been recorded in facilities providing these services [1–8].

NYC’s announcement came 3 years after former NYC Mayor Bill de Blasio first publicly supported OPCs following the release of a feasibility study led by the NYC Department of Health and Mental Hygiene (NYC Health Department) and funded by the New York City Council. The feasibility study assessed the potential impact of incorporating “supervised consumption services” (now referred to as OPC services) into NYC’s overdose response strategy and concluded that the implementation of four NYC OPCs could prevent up to 130 overdose deaths each year.

In 2021, the city renewed its interest in opening OPC services, driven by data showing record-high overdose deaths in NYC. More than 2000 individuals died of a drug overdose in NYC in 2020, the highest number since reporting began in 2000^1^. Following an assessment of existing SSP providers, the city identified OnPoint NYC as having the interest and operational preparedness to move forward with implementing OPC services within their existing Washington Heights and East Harlem SSP facilities. This commentary outlines NYC’s approach to exploring and ultimately supporting OnPoint’s operation of OPCs and documents the lessons learned.

## Purpose

To explore local government’s mechanisms and strategy to support community providers to implement and operate evidence-based OPCs in NYC with the aim of reducing drug overdose deaths and serious injuries.

## Pre-implementation

### Exploring Feasibility

In 2018, NYC conducted a study to assess the feasibility of establishing OPCs in NYC and their projected citywide impact on overdose death rates and public drug use. An analysis of health and fiscal benefits concluded that operating four OPCs in NYC could conservatively prevent up to 130 deaths and save up to $7 million in public health care costs annually. The feasibility study also explored the perspectives of key community partners toward supervised consumption and additionally assessed the current legal landscape to identify possible avenues for implementation.

The sustained and expert advocacy of harm reduction organizations laid the groundwork for the exploration and eventual implementation of OPCs in New York City. Advocacy groups, including VOCAL-NY, the Drug Policy Alliance, and Housing Works, worked for many years to encourage state and federal elected officials to take legislative or executive action to authorize the operation of OPCs statewide and nationwide. Advocacy groups and academic researchers in drug policy, public health, and academic medicine were also instrumental in promoting OPC services as necessary interventions to prevent fatal overdoses in NYC and across the country.

Following the release of the feasibility study, a group of four SSPs in NYC and one SSP in Ithaca, NY, convened to form Research for a Safer New York (RFSNY), a nonprofit entity, to advance the goal of establishing OPCs in New York State (NYS). While RFSNY’s operations slowed and ultimately ceased during the COVID-19 pandemic, the four participating NYC-based SSPs were identified as potential OPC sites^2^.

Although the feasibility study outlined the strong scientific foundation in support of supervised consumption and presented several pathways for opening OPC services, the broader political climate at the time the report was released was not conducive to implementing OPCs—a reality that was further complicated in 2020 by the global pandemic, which impacted government operations at every level. In late 2020, the NYC Health Department reignited its effort to establish OPCs in NYC. With the leadership of then-Mayor Bill de Blasio and in the context of new administrations at the federal and state levels, the city began strategizing around three key domains to facilitate the opening of OPCs: legal and political climate; operational readiness; community engagement. Each domain was approached with several planning questions (Table 1).

**Table 1:** Key planning questions

Legal and political climate	• What are the legal pathways for opening OPCs, and what are the potential legal risks? • Can OPCs operate consistently with local/state/federal law—and without interference from local/state/federal law enforcement and state regulators? • Is there support for OPCs from key political leaders (e.g., local city, state, and federal legislators), and will these leaders/entities publicly state support? If not, what would provide sufficient assurance that they would not interfere with operations?
Operational readiness	• What factors should providers consider before opening and operating an OPC? What support do providers need from local government? • Are providers’ clinical protocols evidence-based and sufficiently robust to encompass a wide array of potential scenarios for participants served? • Do providers need additional funding to operate an OPC, and where will the funding come from? • What is the plan for evaluating the utilization of OPC services? • To what extent have providers engaged the community where the OPCs will be located?
Community engagement	• Who are the key community members/groups to be engaged? • What information should be shared as part of community engagement, and what is the intended outcome of engagement? • How will community concerns be handled when the OPCs open, including press requests and local opposition? How will misinformation be addressed? • What products are needed to get accurate information out to the public about OPCs? • What does continued engagement look like after implementation, including transparency around data, outcomes, and response to problems that arise on the ground?

### Legal and Political Climate

The 2018 feasibility study enumerated the potential pathways for opening OPC services in NYC, including authorization at the federal, state, and local levels. Specifically, the study concluded that state authorization through administrative or legislative action was the most politically feasible and protective against legal challenges. In tandem with the public release of the feasibility study, former Mayor Bill de Blasio announced his support for opening OPCs in NYC, conditional on receiving local district attorney, local council member, and NYS support.

Despite the increasing urgency of the overdose epidemic in New York City and nationwide, the federal political climate at the time of the feasibility report posed significant legal risks to potential OPC providers and their respective jurisdictions, and action was not possible at that time. In 2019, the US Department of Justice (DOJ) took legal action against Safehouse, a nonprofit entity created to operate supervised consumption services in Philadelphia. This action culminated in a Third Circuit Court of Appeals ruling that the proposed OPC violated 21 U.S.C. §856(a), also known as the “crack house statute” of the Controlled Substances Act^3^. In 2021, Safehouse appealed the ruling, and the case is pending a final ruling by DOJ. At the state level, neither administrative nor legislative authorization of OPCs was forthcoming.

In 2021, NY State and the USA elected executive branch leaders who publicly supported harm reduction as a public health approach to reducing overdose deaths. In April 2021, the Biden administration explicitly listed “enhancing evidence-based harm reduction efforts” as a drug policy priority for its first year in office, which NYC interpreted as potentially aligned with the concept of OPCs. Similarly, members of President Biden’s senior leadership team, including Secretary of Health and Human Services Xavier Becerra, voiced harm reduction as a priority, although they did not go so far as to endorse OPCs as a strategy. At the state level, NYS ushered in a new administration in 2021, including newly appointed health leadership who had previously contributed to efforts to explore OPCs in prior roles and was on record as supporting OPCs. Although NYS was not on an immediate path to formally authorize OPCs through executive or legislative action, it appeared that NYS would not interfere if an OPC were to open in NYC.

### Pre-implementation Political Engagement

NYC engaged in a series of discussions with local, state, and federal stakeholders to gauge the viability of opening OPCs in the absence of clear authorization. Strong political engagement of local stakeholders—including the New York City Police Department (NYPD), district attorneys, and local elected officials—was critical to not only mitigate risks of local enforcement against OPC operations but also to ensure successful service provision. Education and engagement of city agencies and elected officials have been ongoing since the release of the feasibility report in 2018. In the ensuing years, the NYC Health Department facilitated multiple visits to OPCs in Europe and Canada to allow local leaders, including senior NYPD officials and some district attorneys, to witness OPC operations and community health and safety impacts. Following the city’s renewed commitment to OPCs in 2021, the NYC Health Department conducted briefings for local elected officials and NYC District Attorneys to secure support for or, at minimum, neutrality toward OPCs.

One significant component of the NYC Health Department’s local political engagement strategy was to consistently advocate for OPCs as the evidence-based, structural response to not only prevent overdose deaths but also reduce public drug use and syringe litter—neighborhood quality of life issues that were particularly salient for community members, local businesses, visitors, elected officials, and city agencies during the summer of 2021. For example, NYC framed OPCs as one intervention to address public drug use in the city’s “joint operations” initiative^4^, a collaboration among the NYC Health Department, Department of Homeless Services, Police Department, health + hospitals, and the Department of Sanitation. By consistently citing the strong evidence base for OPCs, the NYC Health Department was able to develop buy-in across agencies in support of OPCs as an actionable strategy to address the overdose epidemic and reduce public drug use.

In addition to discussions at the local level, extensive engagement with federal and state officials was necessary to assess and mitigate the risk of interference, particularly in the absence of clear endorsements of OPC operations from the federal and state governments. NYC Health Department and the de Blasio administration informed leadership at the NYS Governor’s Office, NYS DOH, and NYS Office of Addiction Services and Supports (OASAS) of NYC’s intention to implement OPCs in NYC as well as federal leaders at Health and Human Services (HHS), Substance Abuse and Mental Health Services (SAMHSA), and the Office of National Drug Control Policy (ONDCP)^5^. Ultimately, in response to the unprecedented number of fatal overdoses reported in 2020, Mayor de Blasio made the decision to endorse OPCs in NYC without explicit support or legal authorization from the federal or state government.

### Operational Readiness

In NYC, OPCs were conceptualized as building upon the programming and operational models of existing SSPs. SSPs offer a range of harm reduction, healthcare, and social services, including but not limited to: syringe exchange, naloxone distribution, drop-in services, safer drug use education, drug checking, and provision of or referrals to substance use disorder treatment, primary care, and mental health care. OPCs are an additional service that can be incorporated into existing SSPs.

The opening of OPC services in NYC would not have been possible without the existence of a publicly supported and funded network of SSPs^6^. Like OPCs, SSPs were a public health intervention with demonstrated effectiveness and strong support among advocates, public health researchers, and people who use drugs. Political opposition hindered the opening of these programs, despite their necessity to address high rates of HIV transmission among people who inject drugs. Furthermore, laws criminalizing the possession of syringes as drug paraphernalia put potential SSP operators and participants at risk of legal consequences. In 1992, SSPs opened in NYC following an emergency authorization by the NYS DOH, demonstrating that public health action in legally murky contexts was not only possible but also necessary to address public health crises. The longstanding relationships of SSPs with the communities they serve and the NYC Health Department, their demonstrable success in reducing the transmission of HIV and other blood-borne illnesses, and their expertise in serving people who use drugs ensured that NYC had providers that were well positioned to open OPC services when it became politically feasible [10].

The four SSPs identified as potential OPC sites all maintain longstanding relationships with the NYC Health Department and have a long history of serving people who use drugs. These SSPs are regulated by the NYS DOH to provide syringe services and receive funding from the NYC Health Department to provide harm reduction services, such as distributing supplies for safer drug use. The readiness of each provider to implement OPC services was considered along the following factors (Table 2).

**Table 2:** Factors for assessing readiness of Syringe Service Providers to implement overdose prevention center services

1) Operations: Space to conduct participant intake, supervised consumption, overdose response, and post-consumption monitoring; competency in services for people who use drugs, including overdose response;
2) Funding: Funding for OPC staff, renovations, and equipment; availability of private funding for all OPC operations as the city determined public funds could not be used for this purpose given the lack of state or federal authorization for OPC services at the time;
3) Approvals: Approval from the landlord of the building housing the site, as well as approval from the SSP’s board of directors/governing body;
4) Acceptance of legal risk and potential backlash: Willingness to accept local support and assurances in lieu of explicit state or federal authorization, as well as potential community backlash, likely in the form of negative press, protests, or community concerns about siting, directed toward the program

OnPoint NYC was the provider most prepared and willing to proceed, in close partnership with the NYC Health Department and Mayor’s Office, toward the goal of incorporating OPC services at their two existing SSP programs.

### Pre-implementation Community Engagement

As with any service provided to the public, NYC Health Department viewed community engagement and education as critical to the success of OPCs, particularly given the stigma that substance use providers and participants often face. Prior to implementation, the NYC Health Department conducted general educational briefings with local community groups and leaders in neighborhoods across the city, including those where the OPCs would be located. This entailed conducting broad public education and engagement about harm reduction as an effective and life-saving approach to drug use and the overdose crisis while incorporating information about OPCs as an additional proven public health strategy to prevent fatal overdoses. Similar to political engagement strategies, materials used for community engagement further emphasized the strong evidence supporting the impact of OPCs in improving public safety and addressing concerns about syringe litter and public drug use.

Through our education efforts, including attendance at Community Board and other community group meetings, the city emphasized the value that an OPC could bring to directly address many of these quality-of-life-related concerns, including syringe litter and public drug use. Furthermore, it was beneficial that OPC services were slated to open in existing SSP facilities, which also house wraparound health and social services, avoiding the need to site a new location. A main component of this education was to ensure we reiterated a few key messages (Table 3).

**Table 3:** Key public messages

□ New York City is in the middle of an overdose crisis. Our friends, neighbors, colleagues, and family members are dying. OPCs save lives
□ In addition to providing a safe place for people to use drugs, OPCs offer personal hygiene facilities, clean clothes, medical and pharmaceutical services, and connections to health care and social services. They are harm reduction hubs that provide connections to vital resources
□ The OPCs are being run by established, trusted, skilled, and regulated professionals in programs that already exist and have ongoing relationships with the communities they serve
□ OPCs are a place-based strategy to reduce overdose deaths in neighborhoods with high overdose burdens. They serve people who reside and spend time in the neighborhoods where they are located. There is no evidence that OPCs draw people who use drugs from outside the neighborhood; on the contrary, research from SSPs demonstrates that most people attend harm reduction services within a 10-min walk of where they live [11]
□ These services also improve community outcomes. Evidence from OPCs worldwide shows that they help reduce public drug use, syringe litter, and drug-related crime

## Implementation

### Local Commitments

Long-term engagement with political stakeholders laid the groundwork for Mayor de Blasio to endorse OPCs as a response to the urgency of the overdose epidemic in the absence of explicit federal or state authorization. Specifically, Mayor de Blasio obtained commitments from local law enforcement and district attorneys that there would be no criminal actions brought against OnPoint NYC or their participants by the city. This provided OnPoint NYC and its Board of Directors confirmation of local government support for OPC operations, including an assurance that local law enforcement would not interfere with the program’s participants or day-to-day operations. An excerpt of this letter from Mayor de Blasio addressed to OnPoint NYC Executive Director, Sam Rivera, is below:

On behalf of the City of New York, I am writing to express our strong support for and commitment to the opening and operation of overdose prevention centers (“OPCs”). OPCs are proven to save lives and have operated safely around the world for decades. It is time for the United States to join countries worldwide that are operating OPCs, and we are proud to help New York City lead the charge.

All city agencies stand ready to ensure the successful launch of OPCs in the five boroughs, which includes a commitment to not take enforcement action against their operation. We have discussed OPCs with relevant law enforcement agencies, including the District Attorneys of the Bronx, Brooklyn, Queens, and Manhattan, and have secured their support for these critical programs as well.

While we are hopeful that the new administrations at both the federal and state levels are supportive of OPCs and expeditiously and publicly affirm that support, we can no longer wait to act at the local level. We stand with you, ready to stop this epidemic and save lives.

### Preparing Operations for Launch

OnPoint NYC developed policies and procedures for OPC services, managed the renovation of their existing space, and secured the required funding. The NYC Health Department met regularly with the program to discuss the strategy for the launch, help formulate an evaluation plan, and provide other guidance as needed. The city was confident in the rigor of OnPoint’s program as outlined in an extensive operational manual that not only described the staffing, space, and equipment requirements to safely operate OPC services but also clearly outlined protocols related to medical and mental health emergency situations. The program’s leadership included staff with direct experience implementing OPC services in Canada. Program leadership’s prior experience in operating OPC services internationally influenced their willingness to be the first publicly recognized OPC provider in the USA and was instrumental in ensuring the successful opening and operations of OPC services in NYC. For example, their prior experience in Canada allowed them to deliver sophisticated overdose response services ranging from naloxone administration to oxygen administration and cardiac response, all of which were documented in comprehensive policies, procedures, and kind of training for staff.

OnPoint NYC expanded the presence of their existing outreach and safety teams in anticipation of OPC’s launch. These teams clean up syringe litter, engage people who use drugs in public and other community members, and direct potential participants to harm reduction services, which would include the OPC once open.

To prepare to evaluate the utilization of OPC services, DOHMH provided technical assistance to develop and refine existing data systems to capture OPC operations. Academic researchers who conducted an evaluation of an unsanctioned OPC in the USA were consulted. The NYC Health Department also identified and funded an academic partner to conduct a multi-year prospective cohort study on OPCs, examining individual and community health outcomes^7^.

## Post-implementation

### Announcement

The city timed the public announcement closely with the beginning of operations. The Mayor’s Office put out a press release on the morning of November 30, 2021, the first day of operations. OnPoint responded to most of the incoming press interviews and brought reporters and community leaders into the site to give them a firsthand look at the OPCs—this went a long way toward dispelling the myths of “drug dens” and other misinformation about operations. NYC Health Department had press staff on site for the first week to field questions and direct press to correct information. Lastly, it was important to show a united front and the need for innovative action to counter the overdose epidemic. On December 15, 2021, BuzzFeed published an op-ed authored by the NYC Health Commissioner and 4 of the 5 borough district attorneys in support of OPCs [12]. OnPoint NYC’s identity was revealed publicly with their permission after the launch of OPC operations.

### Ongoing Community Engagement

Once OPCs were operating in NYC, local Community Board members and other local leaders were invited to tour the sites and see the services firsthand. This has been a powerful tool to demystify OPCs and educate observers about harm reduction. It was helpful, in terms of building community support, that OnPoint already had strong community relationships developed over more than 20 years of operating an SSP. As a result of OnPoint NYC’s consistent community engagement, many community leaders and elected officials have grown to appreciate their work and now serve as strong advocates for OPC services. Some have even called for the expansion of OPC services to other boroughs.

The city also faced opposition from community boards and several advocacy groups in East Harlem and Washington Heights. In East Harlem, in particular, the local community board felt that the opening of an OPC in their community would contribute to an existing “oversaturation” of social and addiction services in the area. Below is an excerpt from a letter to the NYC Health Department, Community Board 11, regarding oversaturation concerns, May 17, 2022:

Like, so many other low-income communities of color, East Harlem has been burdened with hosting more than its fair share of social service facilities, including an oversaturation of mental health and substance abuse treatment and prevention programs. Individuals from not just the five boroughs but as far as Westchester and Long Island participate in programs in our community. This has created a strain on resources and contributed to a range of quality of life and public safety concerns for years on end.

It has remained critical that the NYC Health Department continue to highlight the connection to services/care provided at OPCs, including provision of substance use disorder treatment on-site or through referrals. Since the OPCs opened, briefings for community stakeholders have continued and now include information about the benefits and successes of the OPCs while providing a forum to respond to community questions.

### Current Status

As of this writing, there are two OPCs operating in NYC, with a commitment to expand these services elsewhere in the city. In the first 2 months of operation, the two sites intervened to avert over 125 overdose deaths and severe injuries [13]. Three weeks after the launch of the first publicly recognized overdose prevention centers in the USA, the New York City Board of Health unanimously issued a statement on taking action to prevent drug overdose deaths, urging the federal government to support OPCs. The statement drew particular attention to the connection between evidence-based harm reduction initiatives known to be effective, such as OPCs, and an earlier Board of Health resolution declaring racism as a public health crisis. It also requested that the Health Department and harm reduction providers continue to work together to educate the public and local leaders about the benefit that OPCs offer the community.

## Lessons Learned

This case study aims to share NYC’s experience with OPCs so that other communities across the US may also benefit from this public health intervention. NYC regularly receives questions and requests for discussion from other jurisdictions. To respond, we have summarized four key lessons learned from our experience and prepared the following checklist, which we hope can facilitate these efforts (Table 4). Expansion and sustainability of the OPC model will require sustained advocacy and continued partnership between government and community organizations.

1. Focus on the strong scientific evidence base for OPCs and anticipate key points and sources of opposition.

**Table 4:** Recommended checklist for jurisdictions interested in opening OPCs

Checklist	Examples from NYC
□ Report local data on drug overdose burden	• NYC Health Department publishes yearly Epi Data Briefs on unintentional drug overdose deaths as well reports quarterly data • NYC’s 2018 feasibility study included a literature review summarizing international data on supervised consumption
□ Collect supporting evidence	
□ Determine the readiness of potential OPC providers and their needs (e.g., funding)	• OnPoint is an existing SSP provider with longstanding relationships with the NYC Health Department and a long history of serving people who use drugs • OnPoint developed an operational manual and budget, which were shared with NYC Health Department • OnPoint sought additional public funding for syringe services and private funding for OPC services • NYC Health Department identified and funded an academic partner to conduct a multi-year prospective cohort study on OPCs, examining individual and community health outcomes
□ Plan an evaluation of OPC services	
□ Engage community members, elected officials, and relevant government stakeholders	• NYC Health Department conducted the following engagement: ⚬ Local: Community boards, advocacy groups, city agencies, local elected officials, and district attorneys’ offices ⚬ State: NYS OASAS, NYS DOH, and NYS Governor’s Office ⚬ Federal: HHS, SAMHSA, and ONDCP • BuzzFeed published an op-ed authored by the NYC Health Commissioner and 4 of the 5 borough district attorneys in support of OPCs
□ Proactively identify key stakeholders to validate and voice support	
□ Announce the opening of OPCs along with recent data, sharable assets, and a communications plan	• NYC announced the opening of OPCs via a press release that references 2020 data as well as provisional data for the first quarter of 2021 [9] • After the announcement, NYC Health Department continued to engage community members and elected officials and continued commitment to data transparency. OnPoint continues to host visitors to tour their sites

Over 100 OPCs have operated in Europe and Canada for over 30 years, and the evidence base demonstrating that these services prevent fatal overdose is strong, well documented, and consistent^8^. It will be beneficial to anticipate general questions and common misconceptions, as well as concerns that are specific to the locality and the particular circumstances, and to be able to point to the experience of jurisdictions with comparable experience. To this end, studies demonstrating the role of OPCs in reducing public drug use, syringe litter, and drug-related crime are helpful in addressing community questions and concerns.

2. Identify prospective OPC sites early and deliberately and intensively cultivate relationships between potential program operators and key government players.

Health departments should work with existing harm reduction programs and community-based providers to identify prospective OPC sites. Considerations include whether programs are located in areas with high rates of overdose death, the strength and consistency of programs’ community engagement, and the willingness of programs to operate OPC services in an uncertain legal landscape. Health departments can ensure that political and operational timelines align by garnering support among other government agencies and elected officials, working with programs to develop community and political engagement messaging and strategies, and supporting programs in the development of operational protocols. Centralizing coordination within a health department is critical to ensuring that operational readiness aligns with political will and support to open OPC services.

3. Work to lay the groundwork for political and community support.

Strong prospective OPC providers will likely already have robust, independent, and long-standing relationships with community boards, elected officials, and local businesses and programs. However, health departments should play an active role in creating these relationships where they do not exist. Even when an individual or organization does not explicitly support OPCs, there is still value in moving potential opponents to a position of neutrality. Furthermore, OPCs should be incorporated and consistently referenced in all major official documents, external presentations, and inter-agency discussions as a critical policy response to key community and political concerns, including the overdose crisis, public drug use, and syringe litter.

4. Develop a multipronged communications strategy starting well before launch and extending until after launch.

It is beneficial to develop a communications strategy in collaboration with OPC providers to proactively guide the narrative in the press and among the public. Health departments can assist in identifying key stakeholders within the jurisdiction or beyond to add their voice of support, before and after launch, and to counter narratives that are not evidence-based. By playing a lead role in community engagement and communications, health departments and other city agencies can offer a necessary buffer for the provider, allowing them to focus on program operations. Health departments may partner with potential OPC providers by coordinating tours of OPC sites for the press, elected officials, and key stakeholders to provide an inside view of operations. Lastly, city agencies can amplify OPC data demonstrating the number of overdose deaths and injuries averted and can disseminate evaluation data once ready for publication. We also recommend preparing providers to respond to heightened attention from the press and public.

## Data Availability

Data sharing not applicable to this article as no datasets were generated or analyzed during the current study.

## Appendix: Links to key resources

1. Press release announcing operation of OPC services

2. BuzzFeed Op-Ed by NYC Health Commissioner and District Attorneys

3. 2020 Overdose Death Epi Data Brief

4. 2021 Q1 and Q2 Overdose Death

5. Press release announcing 59 overdoses averted in first three weeks of OPC operation

6. Executive Deputy Commissioner of Mental Hygiene testimony to NYS Senate in support of OPCs

7. NYC Board of Health statement and calls to action and YouTube link to the video

8. NYC Feasibility Study from 2018

9. OPC FAQ

## Footnotes

1 These trends have continued through the first three quarters of 2021. The NYC Health Department expects that the number of overdose deaths in 2021 will exceed those in 2020. [9]

2 Research for a Safer New York (RFSNY) played a critical role not only by advocating at the local, state, and federal levels for OPC authorization, but also by creating a political and community engagement strategy, convening a Scientific Advisory Council to guide the evaluation, and planning for operational protocols.

3 21 U.S.C. §856(a) makes it a felony to knowingly open, lease, rent, use, or maintain any place for the purpose of using any controlled substance.

4 “Joint operations” was a multi-agency initiative aimed to address the intersections of homelessness, public safety, public drug use, quality of life, health, and severe mental illness (SMI) and included hyperlocal outreach to people experiencing homelessness and/or behavioral health needs. The goal was to provide education, awareness, and immediate connection and navigation to health-related services, including OnPoint’s two SSPs. Joint Operations provided the opportunity for agencies to develop a shared understanding of the importance of readily accessible community-based housing, behavioral health, and harm reduction resources as a critical component of successful outreach efforts.

5 In addition to the examples of federal and state stakeholder engagement provided above, NYC Health Department and the de Blasio administration, along with other US cities, penned a letter to US Attorney General Garland calling for action at the federal level to authorize OPCs and asking that DOJ refrain from prosecuting OPCs using the federal Controlled Substances Act. The City publicly called for federal and state authorization via testimony, op-ed, press release, and on-the-record talking points at hearings, press events, and meetings. Local federal law enforcement was also briefed.

6 SSPs opened in NYC in 1992 following emergency authorization by the NYS DOH. The City did not begin funding SSPs until 2005; since then, the NYC Health Department has developed and maintained strong relationships with these programs. The history of SSPs in NYC was highly instructive in the path to opening OPCs.

7 This evaluation aims to identify the impact of OPC utilization on key individual health outcomes, including fatal overdose, nonfatal overdose, emergency department utilization, HIV and hepatitis C transmission, and uptake of treatment and recovery services. Additionally, the study will measure the impacts of OPC operations on the surrounding community, including public drug use, syringe litter, drug-related crime, property values, and local economic activity.

8 Evidence from Vancouver, in particular, demonstrates a significant reduction in overdose deaths in the area immediately surrounding an OPC. Jurisdictions will find peer-reviewed publications using data from OPCs in the US, publicly recognized and unsanctioned, to be particularly helpful in addressing critiques that evidence generated from other countries is not generalizable to the US context [14, 15].

## References

[1] Milloy MS, Kerr T, Tyndall M, Montaner J, Wood E. Estimated drug overdose deaths averted by North America’s first medically-supervised safer injection facility. *PLoS One*. 2008;3(10). 10.1371/journal.pone.0003351 PMC2556397 18839040

[2] Milloy M-J, Kerr T, Mathias R (2008). Non-fatal overdose among a cohort of active injection drug users recruited from a supervised injection facility. Am J Drug Alcohol Abuse.

[3] van Beek I, Kimber J, Dakin A, Gilmour S (2004). The Sydney Medically Supervised Injecting Centre: reducing harm associated with heroin overdose. Crit Public Health.

[4] Kennedy MC, Hayashi K, Milloy M, Wood E, Kerr T. Supervised injection facility use and all-cause mortality among people who inject drugs in Vancouver, Canada: a cohort study. *PLoS Med.* 2019;16(11). 10.1371/journal.pmed.1002964 PMC6879115 31770391

[5] Levengood TW, Yoon GH, Davoust MJ, et al. Supervised injection facilities as harm reduction: a systematic review. *Am J Prev Med*. 2021. 10.1016/j.amepre.2021.04.017 PMC8541900 34218964

[6] Marshall BD, Milloy MJ, Wood E, Montaner JS, Kerr T (2011). Reduction in overdose mortality after the opening of North America’s first medically supervised safer injecting facility: a retrospective population-based study. The Lancet.

[7] Potier C, Laprévote V, Dubois-Arber F, Cottencin O, Rolland B (2014). Supervised injection services: what has been demonstrated? A systematic literature review. Drug Alcohol Depend.

[8] Kral AH, Lambdin BH, Wenger LD, Davidson PJ (2020). Evaluation of an unsanctioned safe consumption site in the United States. N Engl J Med.

[9] *Unintentional drug poisoning (overdose) deaths Quarter 3, 2021, New York City*. NYC Department of Health. June 2022. provisional-overdose-report-third-quarter-2021.pdf(nyc.gov)

[10] Des Jarlais DC, Perlis T, Arasteh K (2005). Reductions in hepatitis C virus and HIV infections among injecting drug users in New York City, 1990–2001. AIDS.

[11] Rockwell R, Des Jarlais DC, Friedman SR, Perlis TE, Paone D (1999). Geographic proximity, policy and utilization of syringe exchange programmes. AIDS Care.

[12] Clark D, Vance C, Gonzalez E, Chokshi D, Katz M. *New York City's new overdose prevention centers have already saved lives*. The Biden Administration Should Get On Board. BuzzFeed News. December 15, 2021. https://www.buzzfeednews.com/article/dadarcelclark/new-york-overdose-prevention-centers. Accessed 10 Jan 2022.

[13] Harocopos A, Gibson BE, Saha N, McRae MT, See K, Rivera S, Chokshi DA (2022). First 2 months of operation at first publicly recognized overdose prevention centers in US. JAMA Netw Open..

